# Pre-treatment mortality and loss-to-follow-up in HIV-1, HIV-2 and HIV-1/HIV-2 dually infected patients eligible for antiretroviral therapy in The Gambia, West Africa

**DOI:** 10.1186/1742-6405-8-24

**Published:** 2011-07-20

**Authors:** Toyin Togun, Ingrid Peterson, Shabbar Jaffar, Francis Oko, Uduak Okomo, Kevin Peterson, Assan Jaye

**Affiliations:** 1 Medical Research Council - The Gambia Unit, Fajara, P.O.Box 273 Banjul, The Gambia; 2 International Centre for AIDS Care and Treatment Programme (ICAP), Swaziland; 3 Department of Epidemiology and Population Health, London School of Hygiene & Tropical Medicine (LSHTM), Keppel Street, London, WC1E 7HT, UK; 4 Institute of Tropical Medicine, Nationalestraat 155, 2000 Antwerp, Belgium; 5 West Africa Platform for HIV Intervention Research (WAPHIR) Network, Universite Cheick Anta Diop, Laboratoire de Bacteriologie Virologie, Hospital A. Le Dantec, Dakar, Senegal

## Abstract

**Background:**

High early mortality rate among HIV infected patients following initiation of antiretroviral therapy (ART) in resource limited settings may indicate high pre-treatment mortality among ART-eligible patients. There is dearth of data on pre-treatment mortality in ART programmes in sub-Sahara Africa. This study aims to determine pre-treatment mortality rate and predictors of pre-treatment mortality among ART-eligible adult patients in a West Africa clinic-based cohort.

**Methods:**

All HIV-infected patients aged 15 years or older eligible for ART between June 2004 and September 2009 were included in the analysis. Assessment for eligibility was based on the Gambia ART guideline. Survival following ART-eligibility was determined by Kaplan-Meier estimates and predictors of pre-treatment mortality determined by Cox proportional hazard models.

**Result:**

Overall, 790 patients were assessed as eligible for ART based on their clinical and/or immunological status among whom 510 (64.6%) started treatment, 26 (3.3%) requested transfer to another health facility, 136 (17.2%) and 118 (14.9%) were lost to follow-up and died respectively without starting ART. ART-eligible patients who died or were lost to follow-up were more likely to be male or to have a CD4 T-cell count < 100 cells/μL, while patients in WHO clinical stage 3 or 4 were more likely to die without starting treatment. The overall pre-treatment mortality rate was 21.9 deaths per 100 person-years (95% CI 18.3 - 26.2) and the rate for the composite end point of death or loss to follow-up was 47.1 per 100 person-years (95% CI 41.6 - 53.2). Independent predictors of pre-treatment mortality were CD4 T-cell count <100 cells/μL (adjusted Hazard ratio [AHR] 3.71; 95%CI 2.54 - 5.41) and WHO stage 3 or 4 disease (AHR 1.91; 95% CI 1.12 - 3.23). Forty percent of ART-eligible patients lost to follow-up seen alive at field visit cited difficulty with the requirement of disclosing their HIV status as reason for not starting ART.

**Conclusion:**

Approximately one third of ART-eligible patients did not start ART and pre-treatment mortality rate was found high among HIV infected patients in our cohort. CD4 T-cell count <100 cells/μL is the strongest independent predictor of pre-treatment mortality. The requirement to disclose HIV status as part of ART preparation counselling constitutes a huge barrier for eligible patients to access treatment.

## Introduction

It is estimated that nearly 37% of people eligible for Antiretroviral therapy (ART) in sub-Sahara Africa were able to access the life saving medicines in 2009 [1]. This increase in availability of ART has, without doubt, impacted positively on the prognosis of HIV/AIDS patients in sub-Sahara Africa with reductions in mortality rate when compared with previous reports in untreated patients [2]. However, data emanating from ART programmes are suggesting that significant programmatic challenges are emerging with the rapid-scale up of ART in low-income settings. While several studies have reported a relatively high mortality rate among patients in the first year of ART initiation [3-8], it has been suggested that the very high mortality rates recorded during the initial months of ART may well reflect a high pre-treatment mortality rate among ART-eligible individuals [9].

There are few data on mortality during the interval between enrolment of ART-eligible HIV-infected adult patients into ART programmes and initiation of treatment in sub-Sahara Africa, and none from the West African region. Information on pre-treatment mortality of ART-eligible HIV infected patients is not routinely collected in most ART programmes in Africa as programme evaluation is most often based on number of patients who start and remain on treatment. The aim of this analysis is to investigate pre-treatment loss to follow-up and to determine mortality rates and predictors of mortality among HIV infected patients who are eligible for ART but have yet to start treatment in the HIV clinic of the Medical Research Council (MRC) The Gambia Unit in Fajara, The Gambia - West Africa.

## Methods

The prevalence of HIV-1 and HIV-2 in The Gambia was estimated to be 1.6% and 0.4% respectively from the 2008 national sentinel surveillance [10], and ART coverage is estimated at 19% [1]. The MRC HIV clinical cohort which started in 1986 enrolled more than 2000 HIV-positive adults on regular active follow-up. Subjects aged 15 yrs or older diagnosed at, or referred to, the HIV clinic of MRC The Gambia Unit were invited to join the sero-prevalent prospective cohort based on an informed consent as previously described [11]. This clinic is situated in an urban area close to the capital city of Banjul, but serves a diverse population of patients including those from rural areas, being a national referral center for HIV care in The Gambia.

At recruitment, data on demographic characteristics, social history and address are collected. A baseline assessment is conducted including determination of clinical stage, complete blood count, biochemistry, chest radiograph and measure of CD4 cell count for all newly diagnosed HIV-positive patients, while routine laboratory monitoring including CD4 T-cell count is done every 6 months or earlier if clinically indicated. Patients are seen at least once every three months by physicians for follow-up clinical assessment and treatment and/or prophylaxis for common opportunistic infections including Cotrimoxazole (Septrin) prophylaxis. The clinic medical team includes a specialist HIV physician and research clinic physicians with at least four years post qualification experience. This clinic uses an electronic registration system and a longitudinal, double-entry data capturing system. All treatments, including antiretroviral therapy, and treatment monitoring are provided free of charge and the patients also receive reimbursement of their transport cost to and from the clinic at every clinic visit including counselling sessions whether on ART or not. Patients who do not come to the clinic for at least 90 days beyond their last scheduled appointment are considered lost to follow-up and are visited at home by trained field workers to ascertain survival status or change of address; if found alive, they are offered reinforced counselling and asked to return to the clinic.

### Selection for Antiretroviral therapy

Combination ART became available in The Gambia in September 2004 through the Global Fund for AIDS, Tuberculosis and Malaria (GFATM), however MRC HIV clinic started providing information on ART to patients in the cohort and assessing them for ART eligibility at the planning phase for introduction of ART in June 2004. Adult patients were considered eligible for ART based on the Gambian National ART guideline [12], which includes: (1) WHO clinical stage 4 disease irrespective of CD4 T-cell count; (2) CD4 T-cell count <200 cells/μL irrespective of clinical stage; or (3) WHO clinical stage 2 or 3 with CD4 cell count<350 cells/μL. Patients already in the cohort with at least a two year history of follow-up were screened by their CD4 T-cell counts and those potentially eligible were offered ART-specific counselling. Although priority for ART was initially given to patients who had been in the cohort for at least two years, this prioritization ended within the first year of ART availability as MRC The Gambia had the capacity to counsel and initiate other eligible patients from the cohort. As a result, assessment for ART became an established clinic practice with other patients offered ART counselling as a part of routine clinic care. The MRC HIV clinic eventually became the largest provider of ART in the country with responsibility for care and follow-up of approximately 50% of all HIV-infected adults on ART in The Gambia.

### Antiretroviral therapy counselling

Antiretroviral therapy counselling, by policy, consists of four one-on-one counselling sessions lasting over three to six weeks, at the end of which patients are required to bring in a treatment supporter who is aware of their HIV-status. Patients who complete the standardized ART-counselling are presented to the national ART Eligibility Committee that meets twice every month for approval to start ART. This committee reviews the patient's clinical and social history and understanding of the pre-ART counselling as assessed by the counsellor.

### Statistical analysis

All HIV infected patients aged 15 years or older assessed as eligible for ART from June 2004 to September 2009 were included in the analysis. Distributions of categorical variables were compared between the four possible outcomes i.e.[a]start treatment; [b] transfer; [c] loss to follow-up; or [d] death by chi square statistics or Fishers exact test as appropriate. Quantitative variables with non-normal distribution were compared using Kruskall-Wallis non-parametric test. For the survival analysis, only patients assessed as eligible for ART up to 30 September 2009 were included and the end of observation period was 30 April 2010. Follow-up duration in person-time was calculated from the date patients were accessed as eligible for ART based on their clinical and/or immunological criteria. Patients who started anti-retroviral therapy were right-censored on the date treatment started. Patients who were lost to follow-up or requested for transfer were followed through to the date of last clinic visit or transfer, respectively. Patients who died were followed to their date of death if known or date last seen alive. Independent risk factors for death were determined by fitting univariate and multivariable Cox proportional hazard regression models. Survival following ART-eligibility was determined and compared between groups using Product-limit (Kaplan-Meier) estimates with log rank tests and summarized by mortality rates reported per 100 person-years. All statistical analyses were done with STATA release 11.1 software (STATA Corp, USA) and statistical significance defined as a p-value of less than 0.05 (2-sided).

## Results

### Baseline characteristics

**Figure 1:**
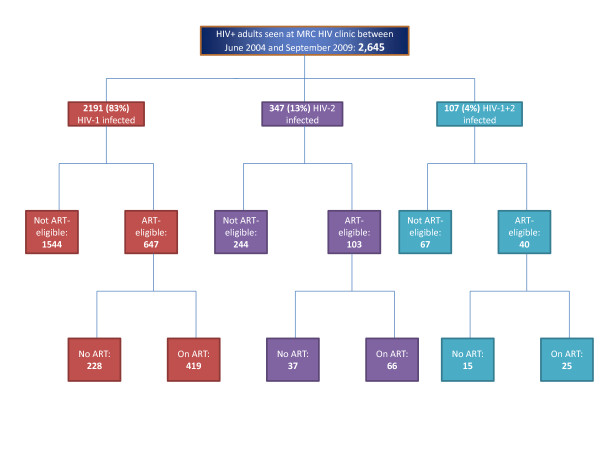
**Profile of the MRC (UK) The Gambia adult HIV Cohort**. Schematic diagram showing the number of HIV infected patients aged ≥15 years seen at the MRC HIV clinic between June 2004 and September 2009. There were 647 HIV-1, 103 HIV-2 and 40 HIV-1/HIV-2 dually infected patients eligible for ART during this period.

Of the 2645 HIV-infected adult patients seen at the MRC HIV clinic between June 2004 and September 2009, 790 were found to be eligible for ART and were enrolled for ART preparation counselling (Figure 1). The median age and median CD4 T-cell count at ART-eligibility was 37 years (IQR: 30 - 44 years) and 150 cells/μL (IQR: 70 - 220 cells/μL) respectively, while 539 patients (68.2%) were female. There were 647 (81.9%) HIV-1, 103 (13.0%) HIV-2 and 40 (5.1%) HIV-1/HIV-2 dually infected patients. Sixty-five (9.0%) of the 723 patients who had WHO clinical staging recorded were in stages 3 or 4, while 217 (45.5%) of 477 with occupation data were unemployed.

Of all the ART-eligible patients, 510 (64.6%) started treatment in our programme, 26 (3.3%) patients voluntarily requested to be transferred to another HIV care facility without starting treatment in our programme, 136 (17.2%) and 118 (14.9%) were lost to follow-up and died respectively without starting treatment. Age, HIV type and occupational status were not significantly associated with the outcomes but there were significant differences by gender, CD4 T cell count and WHO clinical stage (Table 1). ART-eligible patients who were lost to follow-up or died before starting treatment were significantly more likely to be male or have a lower median CD4 T cell count, while patients in WHO stage 3 or 4 are more likely to die before starting treatment.

**Table 1:** Associations of baseline characteristics with the outcomes among 790 ART-eligible patients.

Variable	Started ART	Transferred	LTFU	Died	p-value
**Total (n = 790)**	**510 (64.6)**	**26 (3.3)**	**136 (17.2)**	**118 (14.9)**	
					
**Sex: (n = 790)**					
Male	149 (59.4)	5 (2.0)	48 (19.1)	49 (19.5)	**0.025**^‡^
Female	361 (67.0)	21 (3.9)	88 (16.3)	69 (12.8)	
					
**Age: (n = 790)**					
Median in yrs (IQR)	37 (30, 44)	35 (28, 47)	36 (29, 44)	37 (31, 45)	0.549^§^
<30	116 (62.7)	8 (4.3)	36 (19.5)	25 (13.5)	0.870^‡^
30 - 40	217 (66.1)	10 (3.1)	51 (15.6)	50 (15.2)	
>40	177 (63.9)	8 (2.9)	49 (17.7)	43 (15.5)	
					
**HIV type: (n = 790)**					
HIV-1	419 (64.8)	18 (2.8)	114 (17.6)	96 (14.8)	0.526^Ω^
HIV-2	66 (64.0)	7 (6.8)	15 (14.6)	15 (14.6)	
HIV-dual	25 (62.5)	1 (2.5)	7 (17.5)	7 (17.5)	
					
**CD4 count: (n = 788)**					
Median in cells/ml (IQR)	150 (80, 240)	235 (190, 290)	130 (65, 200)	95 (40, 172)	**0.0001^§^**
<100	149 (57.8)	2 (0.8)	49 (19.0)	58 (22.4)	<**0.001**^‡^
≥100	361 (68.1)	24 (4.5)	87 (16.4)	58 (11.0)	
					
**Occupation: (n = 477)**					
Employed	161 (61.9)	9 (3.5)	45 (17.3)	45 (17.3)	0.074^‡^
Unemployed	154 (71.0)	4 (1.8)	37 (17.1)	22 (10.1)	
					
**WHO stage: (n = 723)**					
1 or 2	425 (64.6)	21 (3.2)	115 (17.5)	97 (14.7)	
3 or 4	33 (50.8)	3 (4.6)	10 (15.4)	19 (29.2)	**0.018**^‡^

Unless otherwise indicated, data are No. (%) of patients in each outcome category

‡Chi-square test

Ω Fishers exact test

§ Kruskall-Wallis test

### Survival analysis

Overall, 118 (14.9%) ART-eligible patients died without starting treatment while 136 (17.2%) were lost to follow-up. The overall pre-treatment mortality rate was 21.9 per 100 person-years (95% CI 18.3 - 26.2), while the rate for the composite endpoint of death or loss to follow-up was 47.1 per 100 person-years (95% CI 41.6 - 53.2). Among patients who started ART, the median interval between ART eligibility and initiation of treatment was 4.3 months. The median survival following ART eligibility for patients who died without starting treatment was 6.7 months with almost all the deaths occurring at home, while the median duration following ART eligibility for patients who were lost to follow-up was 5.4 months.

**Figure 2:**
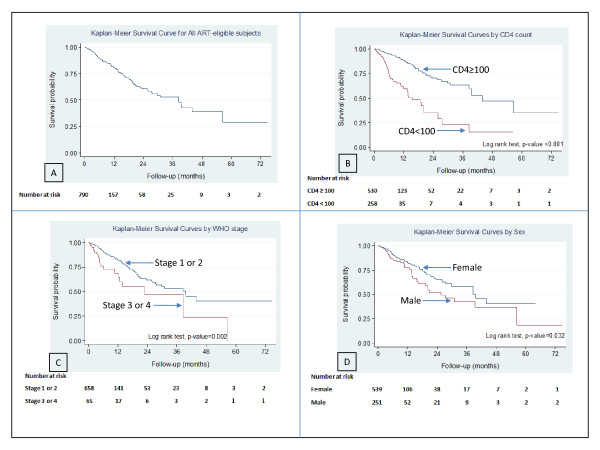
**Survival following eligibility for cART**. Kaplan Meier survival curves with plot A showing survival probability for all ART-eligible patients; while plots B, C, and D shows significant unadjusted associations between CD4 count, WHO clinical stage and Sex respectively, and risks of dying before starting treatment among ART-eligible patients.

In the univariate analysis, male sex, CD4 T-cell count < 100 cells/μL and WHO stage 3 or 4 were all significantly associated with pre-treatment mortality but there was no significant difference in the risk of dying before treatment by HIV type (Table 2). The unadjusted Hazard Ratio for death was 3.69 (95% CI 2.55 - 5.35) for patients with CD4 T-cell count <100 cells/μL compared with≥100 cells/μL. Figure 2 depicts the Kaplan-Meier survival probabilities overall and by baseline CD4 T-cell count, WHO clinical stage at ART eligibility and sex. The graphs show significant unadjusted associations between CD4 T-cell count (log rank test, p < 0.001), WHO clinical stage (log rank test, p = 0.002) and sex (log rank test, p = 0.032) and mortality risk from the time of being assessed as ART-eligible. In the multivariable Cox proportional hazard model, significant independent predictors of pre-treatment mortality were CD4 T-cell count <100 cells/μL and WHO stage 3 or 4. CD4 T-cell count <100 cells/μL is the strongest independent predictor of pre-treatment mortality among ART-eligible patients - the Adjusted Hazard Ratio (AHR)for death was 3.71 (95% CI 2.54 - 5.41) for CD4 T-cell count <100 cells/μL compared to CD4 T-cell count≥100 cells/μL and 1.91 (95% CI 1.12 - 3.23) for being in WHO stage 3 or 4 relative to WHO stage 1 or 2. The proportional hazard assumption is valid in all univariate and the multivariable Cox proportional hazard regression models.

**Table 2:** Univariate and multivariable Cox proportional hazard models of baseline characteristics and risk of pre-treatment mortality in ART-eligible patients^¶^.

		Univariate analysis		Multivariable analysis^Ф^	
		
		Hazard ratio for death (95% CI)	*P*	Hazard ratio for death (95% CI)	*P*
**Sex: (n = 789)**					
Male	251 (31.8%)	1.49 (1.03, 2.15)	**0.036**	1.40 (0.95, 2.07)	0.092
Female	538 (68.2%)	1.0		1.0	
					
**Age: (n = 789)**					
<30	185 (23.5%)	1.0		1.0	
30 - 40	327 (41.4%)	1.10(0.68, 1.78)		1.19 (0.72, 1.98)	
>40	277 (35.1%)	1.09 (0.67, 1.80)	0.914	1.15 (0.67, 1.95)	0.784
					
**HIV type: (n = 789)**					
HIV-1	647 (82.0%)	1.0			
HIV-2	103 (13.1%)	0.73 (0.42, 1.26)		-	
HIV-dual	39 (4.9%)	1.07 (0.49, 2.31)	0.484		
					
**CD4 count: (n = 787)**					
<100	258 (32.8%)	3.69 (2.55, 5.35)	**<0.001**	3.71 (2.54, 5.41)	**<0.001**
≥100	529 (67.2%)	1.0		1.0	
					
**Occupation: (n = 476)**					
Employed	260 (54.6%)	1.0			
Unemployed	216 (45.4%)	0.61 (0.37, 1.02)	0.068	**-**	
					
**WHO stage: (n = 722)**					
1 or 2	657 (91.0%)	1.0		1.0	
3 or 4	65 (9.0%)	2.13 (1.30, 3.49)	**0.006**	1.91 (1.12, 3.23)	**0.017**

¶ Proportional hazard assumption is valid in all univariate and the multivariable Cox proportional hazard models.

Ф Adjusting for Sex, Age, CD4 count category and WHO stage. Occupation was not included in the multivariable model because it was not significant in the univariate analysis; neither does it change the model significantly.

### ART-eligible patients who were lost to follow-up without starting treatment and not known to have died

Home visit of patients who are considered lost to follow-up is done as part of routine clinic practice to ascertain survival status or change of address. Table 3 shows the finding at field visits of the 136 ART-eligible patients who were lost to follow-up without starting treatment and were not known to have died. Forty percent did not complete the ART-counselling because of difficulty in disclosing their HIV status while 10% said they do not want antiretroviral therapy.

**Table 3:** Outcomes of tracing of the 136 ART-eligible patients who were considered lost to follow-up and *not known *to have died

Results	**No**.
Did not complete ART preparation because of "disclosure" issue	54 (40%)
Subject has relocated out of The Gambia	11 (8%)
Receiving care in another health care facility	4 (3%)
Has opted for traditional treatment	4 (3%)
"Don't want Antiretroviral therapy"	14 (10%)
Not found at field visit	35 (26%)
Subject gave no reason but refused to attend clinic	14 (10%)

## Discussion

The reports of high early mortality rate following initiation of ART in resource limited settings [3-8] may indicate high pre-treatment mortality rate. However, there are more reports on clinical outcomes following initiation of treatment in ART programmes in sub-Saharan Africa. Our study reports pre-treatment mortality and losses to follow-up among ART-eligible patients who have yet to start treatment in a West African clinic-based cohort. Overall, 32.1% of all ART-eligible adults from our cohort did not start the potentially life saving treatment. This figure is considerably higher than figures reported from the few available data from southern and East Africa: a retrospective cohort in Durban, South Africa reported 16.4% pre-treatment loss to care [13]; two retrospective cohort studies from Malawi separately reported that 14% and 25.5% of ART-eligible individuals did not start ART [14,15]; and another study from Uganda shows that a quarter of subjects eligible for ART did not initiate on ART [16]. In our cohort, 136 (17.2%) of the 790 ART-eligible patients were lost to follow-up while 118/790 (14.9%) died before treatment could be initiated. The overall pre-treatment mortality rate was 21.9 deaths per 100 person-years with majority of the deaths occurring at home. In this analysis, 26% of patients who were lost to follow-up were 'not found at field visit' and 8% were said to have 'relocated out of The Gambia.' This suggests that our pre-treatment mortality rate is likely to be an underestimate because many of these patients who could not be traced or relocated outside of The Gambia may have died.

The median CD4 T cell count of the ART-eligible individuals is 150 cells/μL indicating that many of the patients were at an advanced stage of immunosuppression by the time they were to start ART. Although ours is a prospective cohort, this observation shows that many of the patients were at an advanced disease stage by the time they were either diagnosed at, or referred to, the MRC HIV clinic especially following the intense nationwide campaign and publicity when ART became available in The Gambia. Those ART-eligible patients who died or were lost to follow-up are more likely to have a low baseline CD4 T cell count compared with those who started treatment or requested to be transferred to another health care facility. Baseline CD4 T cell count <100 cells/μL is the strongest independent predictor of death among ART-eligible subjects in this cohort: patients with CD4 T cell count <100 cells/μL have more than three times the risk of dying before starting treatment compared with those with CD4≥count 100 cells/μL. Therefore, a low baseline CD4 count is not only a risk factor for early mortality following initiation of ART as have been variously reported [3-8], but it is also a significant cause of death among ART-eligible individuals before treatment could be initiated. The median duration of about four months between the time patients are accessed as ART eligible and initiation of treatment is unacceptably long and this probably reflects the time taken for preparation of patients for ART and the need to seek approval from a centralized national eligibility committee before treatment could be started. Given the severity of immunosuppression at baseline, this delay in starting treatment could contribute to pre-treatment mortality as many patients might not be able to complete the counselling session due to ill-health. This further highlights the operational research question about how to balance preparation of ART-eligible patients for life-long ART with the aim for higher survival rate during the process and for optimal treatment success. In addition, there is the need to identify new service delivery models that will increase the uptake of HIV counselling and testing services thereby ensuring earlier diagnosis of HIV infection and initiation of ART.

The WHO clinical stage 3 or 4 was also an independent predictor of pre-treatment mortality in our study. Although studies in routine service delivery settings have reported a low sensitivity of WHO clinical staging in assessing patient's eligibility for ART [17,18], our data suggest that it is an important clinical assessment tool and we therefore advocate for the development of better training materials for ascertainment and diagnosis of common clinical conditions that will contribute to more accurate staging of HIV disease. This will be of importance in the realm of intensified efforts at scaling up access to ART when CD4 T cell counting monitoring is limited in peripheral health centres and there is advocacy for 'task-shifting' of HIV care to allied health care workers [19,20].

Importantly, losses to follow-up following ART-eligibility and risk of death were not correlated in our study to unemployment or lack of economic means to access care (e.g. transportation fare). None of our ART-eligible patients who were lost to follow-up and found alive at field visits cited difficulty with transport cost as a hindrance to seeking care, which reflects the longstanding practice of reimbursement of transport cost of our patients to and from every clinic visitation and the fact that all treatment and investigations are provided free of charge. This finding contrast with the report from Durban, South Africa where unemployed patients were twice as likely to be lost to care before ART initiation in the user-fee paying hospital [13], as well as the report from Uganda where 44% of ART-eligible patients lost to follow-up in this community based clinic did not start ART because they were unable to afford transport cost [16]. Our higher figure of pre-treatment attrition (death and loss to follow-up) when compared to these studies could therefore be attributed more to the operational delivery system and policies that result in delay in access to ART.

The finding that 40% of our ART-eligible patients who were lost to follow-up cited the requirement for 'disclosing' their HIV status to a family member or friend as their reason for not starting ART is of immense significance. This suggests the extent of fear in patients to be rejected and reflects the presence of stigmatization and discrimination against HIV infected persons in the community despite all the many years of awareness and the availability of life saving treatment. Additionally, this figure could likely be an underestimate because a separate 20% of ART-eligible patients lost to follow-up either gave no reason for not completing the ART preparation counselling or stated that they do not want ART. It is our view therefore that greater effort should be put into programmes aimed at countering stigmatization and promoting stronger community participation in ART programmes. Additionally, the need for "disclosure" as a requirement before initiating ART should be revisited or relaxed especially in settings where there might be widespread stigmatization while efforts at countering such are scaled-up.

## Conclusion

Our study that was conducted in the largest ART delivery centre in The Gambia has revealed that a high proportion (one third) of ART-eligible HIV infected patients were not able to start ART and accounted for a high pre-treatment mortality rate. The fact that many of the ART eligible patients had low CD4 T cell counts and that advanced WHO clinical stage was a predictor of death justifies the recent revision of the criteria for ART eligibility aimed at earlier initiation of treatment in HIV infected persons in resource limited settings [21]. At the national level however, there is the need to refocus on a strengthened delivery system that will minimize delays in initiation of ART and help mitigate the clinical, social and economic barriers to accessing ART among eligible HIV infected patients.

## Competing interests

The authors declare that they have no competing interests.

## Authors' contributions

TT co-ordinated the HIV clinical and field activities, performed the statistical analysis and drafted the manuscript. IP contributed to the design of the study. SJ conceived of the idea and participated in the study design. TT, FO, UO and KP participated in the clinical care of the patients. AJ gave overall support and helped to draft the manuscript. All authors read and approved the final manuscript.
